# Supplementary material for: Identification of photoperiod-induced specific miRNAs in the adrenal glands of Sunite sheep (Ovis aries)
Source: Front Vet Sci. 2022 Jul 22;9:888207. doi: 10.3389/fvets.2022.888207 (PMC9354845; doi:10.3389/fvets.2022.888207)
Supplement: Supplementary file 1 [file Data_Sheet_1.ZIP › Supplementary file/miRDeep2 file.zip!/miRDeep2 file/Know_Structure/LP42A1/oar-mir-107.pdf]

miRBase precursor : oar-mir-107
Total read count : 7017
oar-miR-107 read count : 7011
remaining reads : 6

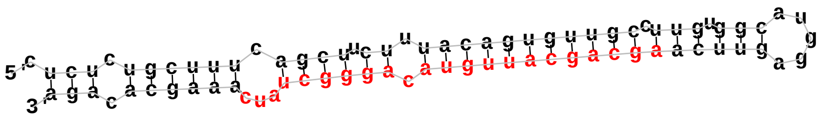

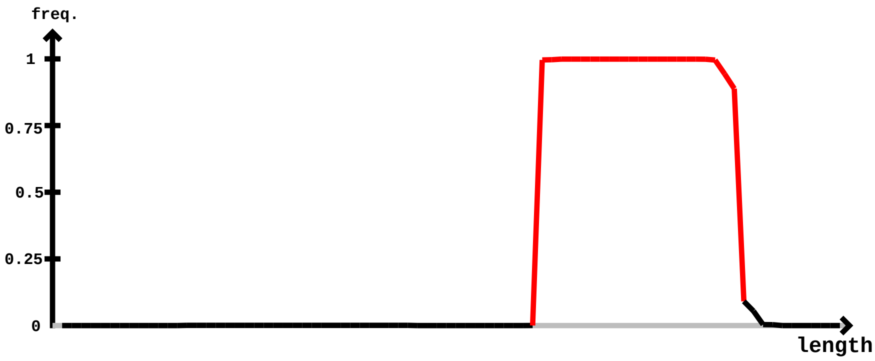

oar-miR-107

5'- cucucugcuuucagcuucuuuacaguguugccuugguggcauggaguucaagcagcauuguacagggcuaucaaagcacaga	-3'	exp		
.(((.((((((.((((.((.(((((((((((.(((.(((.....)))))))))))))))))).))))))...)))))).)))		reads	mm	sample
...........agcuucuuuacagugCugccuugu...		5	1	seq
...........agcuucuuuacaguguugccuugu...		1	0	seq
....aagcagcauuguacagggcuau...........		2	0	seq
....agcagcauuguacaggg...............		3	0	seq
....agcagcauuguacagggc..............		22	0	seq
....agcagcauuguacagggcu.............		366	0	seq
....agcagcauuguacagggcua............		387	0	seq
....agcagcauuguacagggcuau...........		5576	0	seq
....agcagcauuguacagggcuauc..........		255	0	seq
....agcagcauuguacagggcuauca.........		356	0	seq
....agcagcauuguacagggcuaucaa........		3	0	seq
....agcagcauuguacagggcuauGaaa.......		19	1	seq
....gcagcauuguacagggcuau...........		6	0	seq
....cagcauuguacagggcuau...........		11	0	seq
....cagcauuguacagggcuauc..........		1	0	seq
....cagcauuguacagggcuauGaaa.......		4	1	seq
